# Regularity of the edge ideals of perfect [*ν*,*h*]-ary trees and some unicyclic graphs

**DOI:** 10.1016/j.heliyon.2024.e30689

**Published:** 2024-05-08

**Authors:** Fatima Tul Zahra, Muhammad Ishaq, Sarah Aljohani

**Affiliations:** a School of Natural Sciences, National University of Sciences and Technology, Sector H-12, Islamabad, Pakistan; b Department of Mathematics and Sciences, Prince Sultan University, Riyadh 11586, Saudi Arabia

**Keywords:** primary, 13C15, secondary, 13P10, 13F20, Monomial ideal, Edge ideal, Regularity, Perfect [ν,h]-ary tree, Unicyclic graph

## Abstract

We compute the Castelnuovo-Mumford regularity of the quotient rings of edge ideals of perfect [ν,h]-ary trees and some unicyclic graphs.

## 1 Introduction

Let $Z=K[z_{1},\ldots ,z_{h}]$ be a standard graded polynomial ring over a field *K*. Let *Q* be a finitely generated graded *Z*-module. Suppose that *Q* admits the following minimal free resolution:

$$0⟶\underset{g\in Z}{⨁}Z{\left(-g\right)}^{\beta_{q,g}(Q)}⟶\underset{g\in Z}{⨁}Z{\left(-g\right)}^{\beta_{q-1,g}(Q)}⟶\ldots ⟶\underset{g\in Z}{⨁}Z{\left(-g\right)}^{\beta_{0,g}(Q)}⟶Q⟶\;0.$$

The numbers $\beta_{i,g}(Q)$ are (i,g)-th graded Betti numbers and are uniquely determined by *Q*. The *Castelnuovo-Mumford regularity* (or just, theregularity) is an algebraic invariant associated with this minimal graded free resolution, and is defined as follows:

$$\mathrm{reg}(Q)=\max\{g-i:\beta_{i,g}(Q)\neq 0\}.$$

The regularity was introduced by Mumford, generalizing the geometric idea of Castelnuovo [1]. It is quite difficult to find regularity in general by computing graded Betti numbers. For a detailed study on the regularity of monomial ideals and some interesting results we refer the readers to [2], [3], [4], [5], [6], [7], [8], [9], [10], [11]. Let G=(V(G),E(G)) be a finite simple graph, with vertex set $V(G)=\{z_{1},\ldots ,z_{h}\}$ and edge set E(G). An *edge ideal* of a graph G is a squarefree monomial ideal of the polynomial ring *Z* defined as $I(G)=(z_{i}z_{j}:\{z_{i},z_{j}\}\in E(G))$. Villarreal first introduced the idea of edge ideals [12], which become an active research area with a rich literature. In recent years, establishing a relationship between regularity and combinatorial invariants of graph has become a prime focus of many researchers, as evident from [13], [14], [15].

**Figure 1:**
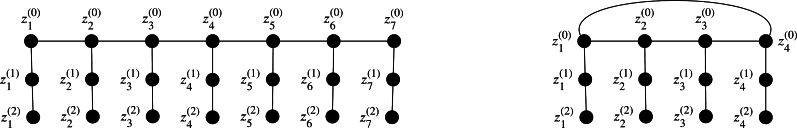
$T_{2,2,7}$ and *C*_2,2,4_, respectively.

**Figure 2:**
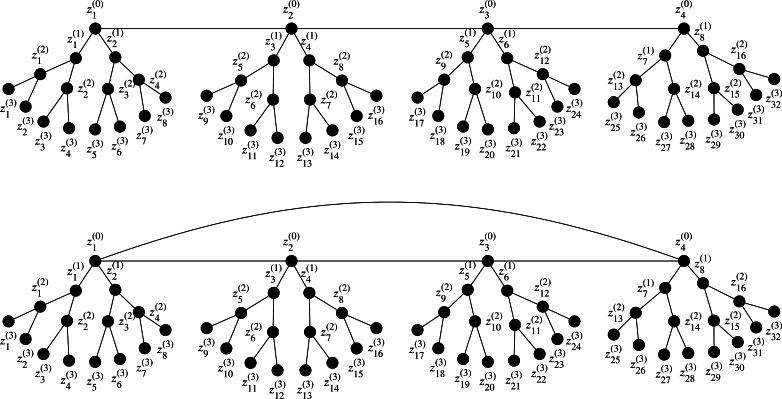
$T_{3,3,4}$ and *C*_3,3,4_, respectively.

The number of vertices incident to a vertex is referred as the *degree* of that vertex. A *pendant vertex* in a graph is a vertex of degree one. A vertex to which an edge is directed from the parent is known as a *child*. A *path* is a graph whose vertices can be ordered so that two vertices are adjacent if and only if they are consecutive in the list. A path graph on *h* vertices is denoted by $P_{h}$. A *tree* is a graph in which there is a unique path between any two vertices of it. A *rooted tree* is a tree in which one vertex is designated as root vertex and all other vertices are directed away from it. A *ν-ary tree* is a tree in which at most *ν* edges are incident to each vertex of this tree. A rooted tree is a *perfect ν-ary tree* if each of its parent vertex has *ν* children, and all non parent vertices are at the same distance from the root vertex. Shaukat et al. [16] calculated the exact values of some algebraic invariants of cyclic modules associated with the edge ideals of perfect *ν*-ary trees. Then, Ayesha et al. defined the perfect [ν,h]-ary trees and some unicyclic graphs closely associated with perfect [ν,h]-ary trees in [17]. Let ν≥2, h≥1 and $W_{1},W_{2},\ldots ,W_{h}$ be perfect (ν−1)-ary trees such that $W_{i}≅W_{j}$, for all *i* and *j*. Let $z_{1},z_{2},\ldots ,z_{h}$ be the root vertices of $W_{1},W_{2},\ldots ,W_{h}$ respectively and $w_{1},w_{2},\ldots ,w_{h}$ be the vertices of path $P_{h}$. Let W be a forest with h+1 components $W_{1},W_{2},\ldots ,W_{h}$ and $P_{h}$. A perfect [ν,h]-ary tree is obtained by fusing the root vertices $z_{i}$ of $W_{i}$ with vertices of path graph $w_{i}$, for all i∈{1,2,…,h} in W. If *d* is the common height of all perfect (ν−1)-ary trees in W then the obtained perfect [ν,h]-ary tree is denoted by $T_{\nu ,d,h}$. Now let h≥3, a unicyclic graph $C_{\nu ,d,h}$ is obtained from $T_{\nu ,d,h}$ by adding an edge between $z_{1}$ and $z_{h}$. See Fig. 1 and Fig. 2 for examples and labelling of $T_{\nu ,d,h}$ and $C_{\nu ,d,h}$. Ayesha et al. calculated some algebraic invariants of the edge ideal of these graphs in [17]. In this article we compute the regularity of the quotient rings of the edge ideals of perfect [ν,h]-ary trees. These results are given in Theorem 3.3 and Theorem 3.5. We also compute the regularity of the quotient rings associated to the edge ideals of $C_{\nu ,d,h}$. For these results, see our Theorem 3.6 and Theorem 3.7.

## 2 Preliminaries

In a graph G, the *neighbourhood* of the vertex $z_{i}$ is the set $N(z_{i}):=\left\{z_{j}:\{z_{i},z_{j}\}\in E(G)\right\}$. If A⊆V(G), then G﹨A denotes the subgraph of G obtained by deleting all vertices of *A* from G and all incident edges on vertices of *A*. If A⊆V(G), then a subgraph $G^{\prime }$ of G is an *induced subgraph* on *A* if $V(G^{\prime })=A$ and $E(G^{\prime })=\left\{\{z_{i},z_{j}\}\in E(G):\{z_{i},z_{j}\}\subseteq A\right\}$. For a graph G, a subset H⊆E(G) is a *matching* in G if all edges in *H* are pairwise non-adjacent. If a set of edges, say $H^{\prime }\subseteq E(G)$, forms a matching in G and is precisely the set of edges of an induced subgraph of G, then that set of edges is referred to as *induced matching*. The maximum cardinality among all possible induced matchings, is called *induced matching number* of G, and denoted as indmat(G).

Remark 2.1 Note that for some specific values of *d* and *h*, a perfect [ν,h]-ary tree $T_{\nu ,d,h}$ reduces to some well-known graphs, for instance:

(1) If ν≥2, d≥1 and h=1, then $T_{\nu ,d,1}$ is a perfect (ν−1)-ary tree.
(2) If ν≥2, d=1 and h≥1, then $T_{\nu ,1,h}$ belongs to the class of caterpillar trees.
(3) If ν≥2, d=2 and h≥1, then $T_{\nu ,2,h}$ belongs to the class of lobster trees.

The values of regularity calculated for path graphs, some caterpillar trees and unicyclic graphs by different authors are given below. These values are helpful in the computation of regularity of perfect [ν,h]-ary trees and the unicyclic graphs we considered. We will use these results in the proofs of our main Theorems.

Lemma 2.2 **[18, Theorem 2.28]**

*Let*ν≥2*and*h≥1*. Then*$\mathrm{reg}(Z/I(T_{\nu ,1,h}))=\left\lceil \frac{h}{2}\right\rceil$*.*

Lemma 2.3 **[18, Theorem 2.30]**

*Let*ν≥2*and*h≥3*. Then*$\mathrm{reg}(Z/I(C_{\nu ,1,h}))=\left\lceil \frac{h-1}{2}\right\rceil$*.*

Lemma 2.4 **[19, Proposition 3.1.1]**

*If*h≥2*, then*$\mathrm{reg}(Z/I(P_{h}))=\left\lceil \frac{h-1}{3}\right\rceil$*.*

Now, we state some results that are frequently used in this article.

Lemma 2.5 **[20, Lemma 2.2]**

*Let*G*be a finite simple graph. Then*

reg(Z/I(G))≥indmat(G).

Lemma 2.6 **[21, Theorem 4.7]**

*Let J be a monomial ideal and z a variable of Z. Then*

(*a*) reg(Z/J)=reg(Z/(J:z))+1*, if* reg(Z/(J:z))>reg(Z/(J,z))*,*

(*b*) reg(Z/J) ∈ {reg(Z/(J,z))+1,reg(Z/(J,z))}*, if* reg(Z/(J:z))=reg(Z/(J,z))*,*

(*c*) reg(Z/J) = reg(Z/(J,z))*, if* reg(Z/(J:z)) < reg(Z/(J,z))*.*

Lemma 2.7 **[22, Theorem 1.2]**

*Let*G*be a unicyclic graph with cycle*$C_{h}$*. Then,*reg(Z/I(G))=indmat(G)+1*if and only if*h≡2(mod3)*and*$\mathrm{indmat}(G﹨N(C_{h}))=\mathrm{indmat}(G)$*, where*$G﹨N(C_{h})$*is the induced subgraph on*$V(G)﹨N(C_{h})$*and*$N(C_{h})$*are the vertices in the neighbourhood of*$C_{h}$*.*

Lemma 2.8 **[23, lemma 8]**

*Let*1≤s<h*. If*$Z_{1}=K[z_{1},\ldots ,z_{s}]$*and*$Z_{2}=K[z_{s+1},\ldots ,z_{h}]$*are rings of polynomial and*$J_{1}$*and*$J_{2}$*are edge ideals of*$Z_{1}$*and*$Z_{2}$*, respectively, then*

$$\mathrm{reg}(Z/(J_{1}Z+J_{2}Z))=\mathrm{reg}(Z_{1}/J_{1})+\mathrm{reg}(Z_{2}/J_{2}).$$

## 3 Regularity of cyclic module associated with perfect [ν,h]-ary tree

Let *d*, *h* and *ν* be integers such that ν≥2 and *d*, h≥1. Let $Q_{\nu ,d,h}:=K[V(T_{\nu ,d,h})]/I(T_{\nu ,d,h})$ and $Q_{\nu ,d,h}^{n}:=\underset{j=1}{\overset{n}{{⊗}_{K}}}\;Q_{\nu ,d,h}$ be the *K*-algebra which is the tensor product of *n* copies of the *K*-algebra $Q_{\nu ,d,h}$ over *K*. In the following remark we address some special cases of $Q_{\nu ,d,h}$ that will be encountered in the proofs of our main theorems.

Remark 3.1 *For our convenience we set* $Q_{\nu ,d,0}=K$ *,* $\mathrm{reg}(Q_{\nu ,d,0})=\mathrm{reg}(K)=0$ *. If we define* $I(P_{1})=(0)$ *then* $\mathrm{reg}(K[V(P_{1})]/I(P_{1}))=\mathrm{reg}(K[z_{h}^{\left(2\right)}])=0$ *. We also define* $Q_{\nu ,0,1}^{{\left(\nu -1\right)}^{2}}=K[z_{(h-2){\left(\nu -1\right)}^{2}+1}^{\left(2\right)},\ldots ,z_{(h-1){\left(\nu -1\right)}^{2}}^{\left(2\right)}]$ *and* $\mathrm{reg}(Q_{\nu ,0,1}^{{\left(\nu -1\right)}^{2}})=0$ *.*

Remark 3.2 *Let* ν≥2 *and d,* h≥1 *. Then by* Lemma 2.8 *we have* $\mathrm{reg}(Q_{\nu ,d,h}^{n})=n\mathrm{reg}(Q_{\nu ,d,h})$ *.*

Theorem 3.3 *Let d,* h≥1 *. Then*

$$\mathrm{reg}(Q_{2,d,h})=\left\{ \begin{array}{cc} \frac{hd}{3}+\left\lceil \frac{h-1}{3}\right\rceil , & d\equiv 0(\mathrm{mod}\;3);\\ h\left\lceil \frac{d}{3}\right\rceil -\left\lfloor \frac{h}{2}\right\rfloor , & d\equiv 1(\mathrm{mod}\;3);\\ h\left\lceil \frac{d}{3}\right\rceil , & d\equiv 2(\mathrm{mod}\;3). \end{array}\right.$$

Proof If h=1, then $Q_{2,d,1}≅K[V(P_{d+1})]/I(P_{d+1})$ and $\mathrm{reg}(Q_{2,d,1})=\mathrm{reg}(K[V(P_{d+1})]/I(P_{d+1}))$, therefore, by Lemma 2.4, we get $\mathrm{reg}(Q_{2,d,1})=\left\lceil \frac{d}{3}\right\rceil$. If h=2, $Q_{2,d,2}≅K[V(P_{2(d+1)})]/I(P_{2(d+1)})$, then again by Lemma 2.4, we have

$$\mathrm{reg}(Q_{2,d,2})=\mathrm{reg}(K[V(P_{2(d+1)})]/I(P_{2(d+1)}))=\left\lceil \frac{2(d+1)-1}{3}\right\rceil =\left\lceil \frac{2d+1}{3}\right\rceil .$$

Now if d≡0(mod3), d=3k, $\mathrm{reg}(Q_{2,d,2})=\left\lceil \frac{6k+1}{3}\right\rceil =\left\lceil 2k+\frac{1}{3}\right\rceil =2k+\left\lceil \frac{1}{3}\right\rceil =\frac{2d}{3}+1$, if d≡1(mod3), d=3k+1, $\mathrm{reg}(Q_{2,d,2})=\left\lceil \frac{6k+3}{3}\right\rceil =2k+1=2\left\lceil \frac{d-1}{3}\right\rceil +1=2(\left\lceil \frac{d}{3}\right\rceil -1)+1=2\left\lceil \frac{d}{3}\right\rceil -1$ and if d≡2(mod3), d=3k+2, $\mathrm{reg}(Q_{2,d,2})=\left\lceil \frac{6k+5}{3}\right\rceil =2k+\left\lceil \frac{5}{3}\right\rceil =2\left\lceil \frac{d-2}{3}\right\rceil +2=2(\left\lceil \frac{d}{3}\right\rceil -1)+2=2\left\lceil \frac{d}{3}\right\rceil$, as required. Now let h≥3 and $Z=K[V(T_{2,d,h})]$. If d=1, the result follows from Lemma 2.2, $\mathrm{reg}(Q_{2,1,h})=\left\lceil \frac{h}{2}\right\rceil$. Now let d≥2, we have the following isomorphisms:

(3.1) $$Z/(I(T_{2,d,h}):z_{h-1}^{\left(0\right)})≅Q_{2,d,h-3}{⊗}_{K}\underset{i=1}{\overset{2}{{⊗}_{K}}}K[V(P_{d})]/I(P_{d}){⊗}_{K}K[V(P_{d-1})]/I(P_{d-1}){⊗}_{K}K[z_{h-1}^{\left(0\right)}],\;$$

and

(3.2) $$Z/(I(T_{2,d,h}),z_{h-1}^{\left(0\right)})≅Q_{2,d,h-2}{⊗}_{K}K[V(P_{d})]/I(P_{d}){⊗}_{K}K[V(P_{d+1})]/I(P_{d+1}).\;$$

**Case 1**: Let d≡2(mod3). Using Lemma 2.8, Lemma 2.4 and Equations (3.1), (3.2), and applying induction on *h* we have

$$\mathrm{reg}\;\left(Z/(I(T_{2,d,h}):z_{h-1}^{\left(0\right)})\right)=\mathrm{reg}(Q_{2,d,h-3})+\sum_{i=1}^{2}\mathrm{reg}(K[V(P_{d})]/I(P_{d}))+\mathrm{reg}(K[V(P_{d-1})]/I(P_{d-1}))\;+\mathrm{reg}(K[z_{h-1}^{\left(0\right)}])\;=(h-3)\left\lceil \frac{d}{3}\right\rceil +2\left\lceil \frac{d-1}{3}\right\rceil +\left\lceil \frac{d-2}{3}\right\rceil \;=(h-1)\left\lceil \frac{d}{3}\right\rceil +\left\lceil \frac{d-2}{3}\right\rceil \;=(h-1)\left\lceil \frac{d}{3}\right\rceil +\left(\left\lceil \frac{d}{3}\right\rceil -1\right)\;=h\left\lceil \frac{d}{3}\right\rceil -1,$$

and

$$\mathrm{reg}\;\left(Z/(I(T_{2,d,h}),z_{h-1}^{\left(0\right)})\right)=\mathrm{reg}(Q_{2,d,h-2})+\mathrm{reg}(K[V(P_{d})]/I(P_{d}))+\mathrm{reg}(K[V(P_{d+1})]/I(P_{d+1}))\;=(h-2)\left\lceil \frac{d}{3}\right\rceil +\left\lceil \frac{d-1}{3}\right\rceil +\left\lceil \frac{d}{3}\right\rceil \;=h\left\lceil \frac{d}{3}\right\rceil .$$

Clearly, $\mathrm{reg}\;\left(Z/(I(T_{2,d,h}),z_{h-1}^{\left(0\right)})\right)>\mathrm{reg}\;\left(Z/(I(T_{2,d,h}):z_{h-1}^{\left(0\right)})\right)$. Hence, by using Lemma 2.6 (*c*), $\mathrm{reg}(Q_{2,d,h})=h\left\lceil \frac{d}{3}\right\rceil$.

**Case 2 :** Let d≡0(mod3). Using Lemma 2.8, Lemma 2.4 and Equations (3.1), (3.2), and applying induction on *h* we have

$$\mathrm{reg}\;\left(Z/(I(T_{2,d,h}):z_{h-1}^{\left(0\right)})\right)=\mathrm{reg}(Q_{2,d,h-3})+\sum_{i=1}^{2}\mathrm{reg}(K[V(P_{d})]/I(P_{d}))\;+\mathrm{reg}(K[V(P_{d-1})]/I(P_{d-1}))+\mathrm{reg}(K[z_{h-1}^{\left(0\right)}])\;=(h-3)\frac{d}{3}+\left\lceil \frac{h-4}{3}\right\rceil +2\left\lceil \frac{d-1}{3}\right\rceil +\left\lceil \frac{d-2}{3}\right\rceil ,$$

and

$$\mathrm{reg}\;\left(Z/(I(T_{2,d,h}),z_{h-1}^{\left(0\right)})\right)=\mathrm{reg}(Q_{2,d,h-2})+\mathrm{reg}(K[V(P_{d})]/I(P_{d}))+\mathrm{reg}(K[V(P_{d+1})]/I(P_{d+1}))\;=(h-2)\frac{d}{3}+\left\lceil \frac{h-3}{3}\right\rceil +\left\lceil \frac{d-1}{3}\right\rceil +\left\lceil \frac{d}{3}\right\rceil .$$

Since d≡0(mod3) implies $\left\lceil \frac{d-1}{3}\right\rceil =\left\lceil \frac{d-2}{3}\right\rceil =\frac{d}{3}$. So we have

$$\mathrm{reg}\;\left(Z/(I(T_{2,d,h}):z_{h-1}^{\left(0\right)})\right)=\frac{hd}{3}+\left\lceil \frac{h-4}{3}\right\rceil ,$$

and

$$\mathrm{reg}\;\left(Z/(I(T_{2,d,h}),z_{h-1}^{\left(0\right)})\right)=\frac{hd}{3}+\left\lceil \frac{h-3}{3}\right\rceil .$$

If h≡0(mod3), then $\left\lceil \frac{h-4}{3}\right\rceil =\left\lceil \frac{h-3}{3}\right\rceil$, so we have

$$\mathrm{reg}\;\left(Z/(I(T_{2,d,h}):z_{h-1}^{\left(0\right)})\right)=\mathrm{reg}\;\left(Z/(I(T_{2,d,h}),z_{h-1}^{\left(0\right)})\right).$$

Thus, by using Lemma 2.6 (*b*),

$$\mathrm{reg}(Q_{2,d,h})\in \left\{\mathrm{reg}\;\left(Z/(I(T_{2,d,h}),z_{h-1}^{\left(0\right)})\right)+1,\mathrm{reg}\;\left(Z/(I(T_{2,d,h}),z_{h-1}^{\left(0\right)})\right)\right\}\mathrm{reg}(Q_{2,d,h})\in \left\{\frac{hd}{3}+\left\lceil \frac{h-4}{3}\right\rceil +1,\frac{hd}{3}+\left\lceil \frac{h-4}{3}\right\rceil \right\}\mathrm{reg}(Q_{2,d,h})\leq \frac{hd}{3}+\left\lceil \frac{h-1}{3}\right\rceil .$$

If h≡1(mod3), then $\left\lceil \frac{h-3}{3}\right\rceil =\left\lceil \frac{h-1}{3}\right\rceil$ and $\left\lceil \frac{h-1}{3}\right\rceil >\left\lceil \frac{h-4}{3}\right\rceil$. So we have

$$\mathrm{reg}\;\left(Z/(I(T_{2,d,h}),z_{h-1}^{\left(0\right)})\right)>\mathrm{reg}\;\left(Z/(I(T_{2,d,h}):z_{h-1}^{\left(0\right)})\right).$$

Thus, by using Lemma 2.6 (*c*), we have $\mathrm{reg}(Q_{2,d,h})=\frac{hd}{3}+\left\lceil \frac{h-1}{3}\right\rceil$.

If h≡2(mod3), then $\left\lceil \frac{h-4}{3}\right\rceil =\left\lceil \frac{h-3}{3}\right\rceil$, so we have

$$\mathrm{reg}\;\left(Z/(I(T_{2,d,h}),z_{h-1}^{\left(0\right)})\right)=\mathrm{reg}\;\left(Z/(I(T_{2,d,h}):z_{h-1}^{\left(0\right)})\right).$$

Thus, by using Lemma 2.6 (*b*), we have

$$\mathrm{reg}(Q_{2,d,h})\in \left\{\mathrm{reg}\;\left(Z/(I(T_{2,d,h}),z_{h-1}^{\left(0\right)})\right)+1,\mathrm{reg}\;\left(Z/(I(T_{2,d,h}),z_{h-1}^{\left(0\right)})\right)\right\}\mathrm{reg}(Q_{2,d,h})\in \left\{\frac{hd}{3}+\left\lceil \frac{h-4}{3}\right\rceil +1,\frac{hd}{3}+\left\lceil \frac{h-4}{3}\right\rceil \right\}\mathrm{reg}(Q_{2,d,h})\leq \frac{hd}{3}+\left\lceil \frac{h-1}{3}\right\rceil .$$

Now for finding lower bound when h≡0,2(mod3) we find an induced matching of $T_{2,d,h}$ as follows:

$$E_{1}=\overset{h}{\underset{i=1}{⋃}}\left\{\{z_{i}^{\left(d-1\right)},z_{i}^{\left(d\right)}\}\right\},E_{2}=\overset{h}{\underset{i=1}{⋃}}\left\{\{z_{i}^{\left(d-4\right)},z_{i}^{\left(d-3\right)}\}\right\},\cdots ,E_{\frac{d}{3}}=\overset{h}{\underset{i=1}{⋃}}\left\{\{z_{i}^{\left(2\right)},z_{i}^{\left(3\right)}\}\right\}.$$

Clearly $|E_{i}|=h$, for all *i*. Let 1≤j≤h−1, $I_{j}=\left\{\{z_{j}^{\left(0\right)},z_{j+1}^{\left(0\right)}\}\right\}$ be the edges of graph $T_{2,d,h}$. Let $I=\left\{I_{1},I_{4},\ldots ,I_{1+3\left\lceil \frac{h-4}{3}\right\rceil }\right\}$ and $E=E_{1}\cup E_{2}\cup \ldots \cup E_{\frac{d}{3}}\cup I$. It can easily be verified that *E* is an induced matching of $T_{2,d,h}$. Therefore, $\mathrm{indmat}(T_{2,d,h})\geq |E|$, where $|E|=\frac{hd}{3}+\left\lceil \frac{h-1}{3}\right\rceil$. According to Lemma 2.5, we have $\mathrm{reg}(Q_{2,d,h})\geq \frac{hd}{3}+\left\lceil \frac{h-1}{3}\right\rceil$. Hence, $\mathrm{reg}(Q_{2,d,h})=\frac{hd}{3}+\left\lceil \frac{h-1}{3}\right\rceil$, as required.

**Case 3 :** Let d≡1(mod3). Using Lemma 2.8, Lemma 2.4 and Equations (3.1), (3.2), and applying induction on *h* we have

$$\mathrm{reg}\;\left(Z/(I(T_{2,d,h}):z_{h-1}^{\left(0\right)})\right)=\mathrm{reg}(Q_{2,d,h-3})+\sum_{i=1}^{2}\mathrm{reg}(K[V(P_{d})]/I(P_{d}))+\mathrm{reg}(K[V(P_{d-1})]/I(P_{d-1}))\;+\mathrm{reg}(K[z_{h-1}^{\left(0\right)}])\;=(h-3)\left\lceil \frac{d}{3}\right\rceil -\left\lfloor \frac{h-3}{2}\right\rfloor +2\left\lceil \frac{d-1}{3}\right\rceil +\left\lceil \frac{d-2}{3}\right\rceil \;=(h-3)\left\lceil \frac{d}{3}\right\rceil -\left\lfloor \frac{h-3}{2}\right\rfloor +2\left(\left\lceil \frac{d}{3}\right\rceil -1\right)+\left\lceil \frac{d}{3}\right\rceil -1\;=h\left\lceil \frac{d}{3}\right\rceil -\left(\left\lfloor \frac{h-3}{2}\right\rfloor +3\right)\;=h\left\lceil \frac{d}{3}\right\rceil -\left\lfloor \frac{h+3}{2}\right\rfloor ,$$

and

$$\mathrm{reg}\;\left(Z/(I(T_{2,d,h}),z_{h-1}^{\left(0\right)})\right)=\mathrm{reg}(Q_{2,d,h-2})+\mathrm{reg}(K[V(P_{d})]/I(P_{d}))+\mathrm{reg}(K[V(P_{d+1})]/I(P_{d+1}))\;=(h-2)\left\lceil \frac{d}{3}\right\rceil -\left\lfloor \frac{h-2}{2}\right\rfloor +\left\lceil \frac{d-1}{3}\right\rceil +\left\lceil \frac{d}{3}\right\rceil \;=(h-2)\left\lceil \frac{d}{3}\right\rceil -\left\lfloor \frac{h-2}{2}\right\rfloor +\left(\left\lceil \frac{d}{3}\right\rceil -1\right)+\left\lceil \frac{d}{3}\right\rceil \;=h\left\lceil \frac{d}{3}\right\rceil -\left(\left\lfloor \frac{h-2}{2}\right\rfloor +1\right)\;=h\left\lceil \frac{d}{3}\right\rceil -\left\lfloor \frac{h}{2}\right\rfloor .$$

Clearly, $\mathrm{reg}\;\left(Z/(I(T_{2,d,h}),z_{h-1}^{\left(0\right)})\right)>\mathrm{reg}\;\left(Z/(I(T_{2,d,h}):z_{h-1}^{\left(0\right)})\right)$. Hence, by using Lemma 2.6 (*c*), $\mathrm{reg}(Q_{2,d,h})=h\left\lceil \frac{d}{3}\right\rceil -\left\lfloor \frac{h}{2}\right\rfloor$. □

Lemma 3.4 **[16, Proposition 4.4]**

*Let* ν≥3 *and* d≥1 *. Then*

$$\mathrm{reg}(Q_{\nu ,d,1})=\left\{ \begin{array}{cc} \frac{{\left(\nu -1\right)}^{d+2}-{\left(\nu -1\right)}^{2}}{{\left(\nu -1\right)}^{3}-1}, & d\equiv 0(\mathrm{mod}\;3);\\ \frac{{\left(\nu -1\right)}^{d+2}-1}{{\left(\nu -1\right)}^{3}-1}, & d\equiv 1(\mathrm{mod}\;3);\\ \frac{{\left(\nu -1\right)}^{d+2}-\nu +1}{{\left(\nu -1\right)}^{3}-1}, & d\equiv 2(\mathrm{mod}\;3). \end{array}\right.$$

Theorem 3.5 *Let* ν≥3 *and d,* h≥1 *. Then*

$$\mathrm{reg}(Q_{\nu ,d,h})=\left\{ \begin{array}{cc} \frac{h({\left(\nu -1\right)}^{d+2}-{\left(\nu -1\right)}^{2})}{{\left(\nu -1\right)}^{3}-1}+\left\lceil \frac{h-1}{3}\right\rceil , & d\equiv 0(\mathrm{mod}\;3);\\ \frac{h({\left(\nu -1\right)}^{d+2}-1)}{{\left(\nu -1\right)}^{3}-1}-\left\lfloor \frac{h}{2}\right\rfloor , & d\equiv 1(\mathrm{mod}\;3);\\ \frac{h({\left(\nu -1\right)}^{d+2}-(\nu -1))}{{\left(\nu -1\right)}^{3}-1}, & d\equiv 2(\mathrm{mod}\;3). \end{array}\right.$$

Proof If h=1, the result follows from Lemma 3.4. Now, let h≥2 and $Z=K[V(T_{\nu ,d,h})]$. If d=1, the result follows from Lemma 2.2, $\mathrm{reg}(Q_{\nu ,1,h})=\left\lceil \frac{h}{2}\right\rceil$. Let d≥2. If h=2, we have the following isomorphisms:

(3.3) $$Z/(I(T_{\nu ,d,2}):z_{1}^{\left(0\right)})≅Q_{\nu ,d-2,1}^{{\left(\nu -1\right)}^{2}}{⊗}_{K}Q_{\nu ,d-1,1}^{\left(\nu -1\right)}{⊗}_{K}K[z_{1}^{\left(0\right)}],\;$$

and

(3.4) $$Z/(I(T_{\nu ,d,2}),z_{1}^{\left(0\right)})≅Q_{\nu ,d-1,1}^{\left(\nu -1\right)}{⊗}_{K}Q_{\nu ,d,1}.\;$$

Now if h≥3, we have

(3.5) $$Z/(I(T_{\nu ,d,h}):z_{h-1}^{\left(0\right)})≅Q_{\nu ,d,h-3}{⊗}_{K}\underset{i=1}{\overset{2}{{⊗}_{K}}}Q_{\nu ,d-1,1}^{\left(\nu -1\right)}{⊗}_{K}Q_{\nu ,d-2,1}^{{\left(\nu -1\right)}^{2}}{⊗}_{K}K[z_{h-1}^{\left(0\right)}],\;$$

and

(3.6) $$Z/(I(T_{\nu ,d,h}),z_{h-1}^{\left(0\right)})≅Q_{\nu ,d,h-2}{⊗}_{K}Q_{\nu ,d-1,1}^{\left(\nu -1\right)}{⊗}_{K}Q_{\nu ,d,1}.\;$$

We consider the following cases:

**Case 1 :** Let d≡2(mod3). As d−1≡1(mod3) and d−2≡0(mod3), for h=2, using Remark 3.2, Lemma 3.4, Lemma 2.8 and Equations (3.3), (3.4) we have

$$\mathrm{reg}\;\left(Z/(I(T_{\nu ,d,2}):z_{1}^{\left(0\right)})\right)=\mathrm{reg}(Q_{\nu ,d-2,1}^{{\left(\nu -1\right)}^{2}})+\mathrm{reg}(Q_{\nu ,d-1,1}^{\left(\nu -1\right)})+\mathrm{reg}(K[z_{1}^{\left(0\right)}])\;={\left(\nu -1\right)}^{2}\mathrm{reg}(Q_{\nu ,d-2,1})+(\nu -1)\mathrm{reg}(Q_{\nu ,d-1,1})+\mathrm{reg}(K[z_{1}^{\left(0\right)}])\;={\left(\nu -1\right)}^{2}\left(\frac{{\left(\nu -1\right)}^{(d-2)+2}-{\left(\nu -1\right)}^{2}}{{\left(\nu -1\right)}^{3}-1}\right)+(\nu -1)\left(\frac{{\left(\nu -1\right)}^{(d-1)+2}-1}{{\left(\nu -1\right)}^{3}-1}\right)\;=\frac{{\left(\nu -1\right)}^{d+2}-{\left(\nu -1\right)}^{4}}{{\left(\nu -1\right)}^{3}-1}+(\nu -1)+\frac{{\left(\nu -1\right)}^{d+2}-(\nu -1)}{{\left(\nu -1\right)}^{3}-1}-(\nu -1)\;=\frac{2({\left(\nu -1\right)}^{d+2}-(\nu -1))}{{\left(\nu -1\right)}^{3}-1}-(\nu -1),$$

and

$$\mathrm{reg}\;\left(Z/(I(T_{\nu ,d,2}),z_{1}^{\left(0\right)})\right)=\mathrm{reg}(Q_{\nu ,d-1,1}^{\left(\nu -1\right)})+\mathrm{reg}(Q_{\nu ,d,1})\;=(\nu -1)\mathrm{reg}(Q_{\nu ,d-1,1})+\mathrm{reg}(Q_{\nu ,d,1})\;=(\nu -1)\left(\frac{{\left(\nu -1\right)}^{(d-1)+2}-1}{{\left(\nu -1\right)}^{3}-1}\right)+\frac{{\left(\nu -1\right)}^{d+2}-\nu +1}{{\left(\nu -1\right)}^{3}-1}\;=\frac{2({\left(\nu -1\right)}^{d+2}-(\nu -1))}{{\left(\nu -1\right)}^{3}-1}.$$

Now for h≥3, using Remark 3.2, Lemma 3.4, Lemma 2.8 and Equations (3.5), (3.6), and applying induction on *h* we have

$$\mathrm{reg}\;\left(Z/(I(T_{\nu ,d,h}):z_{h-1}^{\left(0\right)})\right)=\mathrm{reg}(Q_{\nu ,d,h-3})+\sum_{i=1}^{2}\mathrm{reg}(Q_{\nu ,d-1,1}^{\left(\nu -1\right)})+\mathrm{reg}(Q_{\nu ,d-2,1}^{{\left(\nu -1\right)}^{2}})+\mathrm{reg}(K[z_{h-1}^{\left(0\right)}])\;=\mathrm{reg}(Q_{\nu ,d,h-3})+\sum_{i=1}^{2}(\nu -1)\mathrm{reg}(Q_{\nu ,d-1,1})+{\left(\nu -1\right)}^{2}\mathrm{reg}(Q_{\nu ,d-2,1})\;+\mathrm{reg}(K[z_{h-1}^{\left(0\right)}])\;=(h-3)\left(\frac{{\left(\nu -1\right)}^{d+2}-\nu +1}{{\left(\nu -1\right)}^{3}-1}\right)+2(\nu -1)\left(\frac{{\left(\nu -1\right)}^{(d-1)+2}-1}{{\left(\nu -1\right)}^{3}-1}\right)\;+{\left(\nu -1\right)}^{2}\left(\frac{{\left(\nu -1\right)}^{(d-2)+2}-{\left(\nu -1\right)}^{2}}{{\left(\nu -1\right)}^{3}-1}\right)\;=(h-1)\left(\frac{{\left(\nu -1\right)}^{d+2}-\nu +1}{{\left(\nu -1\right)}^{3}-1}\right)+\frac{{\left(\nu -1\right)}^{d+2}-{\left(\nu -1\right)}^{4}}{{\left(\nu -1\right)}^{3}-1}+(\nu -1)\;-(\nu -1)\;=\frac{h({\left(\nu -1\right)}^{d+2}-(\nu -1))}{{\left(\nu -1\right)}^{3}-1}-(\nu -1),$$

and

$$\mathrm{reg}\;\left(Z/(I(T_{\nu ,d,h}),z_{h-1}^{\left(0\right)})\right)=\mathrm{reg}(Q_{\nu ,d,h-2})+\mathrm{reg}(Q_{\nu ,d-1,1}^{\left(\nu -1\right)})+\mathrm{reg}(Q_{\nu ,d,1})\;=\mathrm{reg}(Q_{\nu ,d,h-2})+(\nu -1)\mathrm{reg}(Q_{\nu ,d-1,1})+\mathrm{reg}(Q_{\nu ,d,1})\;=(h-2)\left(\frac{{\left(\nu -1\right)}^{d+2}-\nu +1}{{\left(\nu -1\right)}^{3}-1}\right)+(\nu -1)\left(\frac{{\left(\nu -1\right)}^{(d-1)+2}-1}{{\left(\nu -1\right)}^{3}-1}\right)\;+\frac{{\left(\nu -1\right)}^{d+2}-\nu +1}{{\left(\nu -1\right)}^{3}-1}\;=\frac{h({\left(\nu -1\right)}^{d+2}-(\nu -1))}{{\left(\nu -1\right)}^{3}-1}.$$

Clearly for all h≥2, $\mathrm{reg}\;\left(Z/(I(T_{\nu ,d,h}),z_{h-1}^{\left(0\right)})\right)>\mathrm{reg}\;\left(Z/I(T_{\nu ,d,h}):z_{h-1}^{\left(0\right)})\right)$. Hence, by using Theorem 2.6 (*c*), $\mathrm{reg}(Q_{\nu ,d,h})=\frac{h({\left(\nu -1\right)}^{d+2}-(\nu -1))}{{\left(\nu -1\right)}^{3}-1}$.

**Case 2 :** Let d≡0(mod3). As d−1≡2(mod3) and d−2≡1(mod3), for h=2, using Remark 3.2, Lemma 3.4, Lemma 2.8 and Equations (3.3), (3.4) we have

$$\mathrm{reg}\;\left(Z/(I(T_{\nu ,d,2}):z_{1}^{\left(0\right)})\right)=\mathrm{reg}(Q_{\nu ,d-2,1}^{{\left(\nu -1\right)}^{2}})+\mathrm{reg}(Q_{\nu ,d-1,1}^{\left(\nu -1\right)})+\mathrm{reg}(K[z_{1}^{\left(0\right)}])\;={\left(\nu -1\right)}^{2}\mathrm{reg}(Q_{\nu ,d-2,1})+(\nu -1)\mathrm{reg}(Q_{\nu ,d-1,1})+\mathrm{reg}(K[z_{1}^{\left(0\right)}])\;={\left(\nu -1\right)}^{2}\left(\frac{{\left(\nu -1\right)}^{(d-2)+2}-1}{{\left(\nu -1\right)}^{3}-1}\right)+(\nu -1)\left(\frac{{\left(\nu -1\right)}^{(d-1)+2}-(\nu -1)}{{\left(\nu -1\right)}^{3}-1}\right)\;=\frac{2({\left(\nu -1\right)}^{d+2}-{\left(\nu -1\right)}^{2})}{{\left(\nu -1\right)}^{3}-1},$$

and

$$\mathrm{reg}\;\left(Z/(I(T_{\nu ,d,2}),z_{1}^{\left(0\right)})\right)=\mathrm{reg}(Q_{\nu ,d-1,1}^{\left(\nu -1\right)})+\mathrm{reg}(Q_{\nu ,d,1})\;=(\nu -1)\mathrm{reg}(Q_{\nu ,d-1,1})+\mathrm{reg}(Q_{\nu ,d,1})\;=(\nu -1)\left(\frac{{\left(\nu -1\right)}^{(d-1)+2}-(\nu -1)}{{\left(\nu -1\right)}^{3}-1}\right)+\frac{{\left(\nu -1\right)}^{d+2}-{\left(\nu -1\right)}^{2}}{{\left(\nu -1\right)}^{3}-1}\;=\frac{2({\left(\nu -1\right)}^{d+2}-{\left(\nu -1\right)}^{2})}{{\left(\nu -1\right)}^{3}-1}.$$

Thus, by using Lemma 2.2 (*b*), $\mathrm{reg}(Q_{\nu ,d,2})\in \left\{\frac{2({\left(\nu -1\right)}^{d+2}-{\left(\nu -1\right)}^{2})}{{\left(\nu -1\right)}^{3}-1}+1,\frac{2({\left(\nu -1\right)}^{d+2}-{\left(\nu -1\right)}^{2})}{{\left(\nu -1\right)}^{3}-1}\right\}$.

Hence, we have $\mathrm{reg}(Q_{\nu ,d,2})\leq \frac{2({\left(\nu -1\right)}^{d+2}-{\left(\nu -1\right)}^{2})}{{\left(\nu -1\right)}^{3}-1}+1$. Now for h≥3, using Remark 3.2, Lemma 3.4, Lemma 2.8 and Equations (3.5), (3.6), and applying induction on *h* we have

$$\mathrm{reg}\;\left(Z/(I(T_{\nu ,d,h}):z_{h-1}^{\left(0\right)})\right)=\mathrm{reg}(Q_{\nu ,d,h-3})+\sum_{i=1}^{2}\mathrm{reg}(Q_{\nu ,d-1,1}^{\left(\nu -1\right)})+\mathrm{reg}(Q_{\nu ,d-2,1}^{{\left(\nu -1\right)}^{2}})+\mathrm{reg}(K[z_{h-1}^{\left(0\right)}])\;=\mathrm{reg}(Q_{\nu ,d,h-3})+\sum_{i=1}^{2}(\nu -1)\mathrm{reg}(Q_{\nu ,d-1,1})+{\left(\nu -1\right)}^{2}\mathrm{reg}(Q_{\nu ,d-2,1})\;+\mathrm{reg}(K[z_{h-1}^{\left(0\right)}])\;=(h-3)\left(\frac{{\left(\nu -1\right)}^{d+2}-{\left(\nu -1\right)}^{2}}{{\left(\nu -1\right)}^{3}-1}\right)+\left\lceil \frac{h-4}{3}\right\rceil \;+2(\nu -1)\left(\frac{{\left(\nu -1\right)}^{(d-1)+2}-(\nu -1)}{{\left(\nu -1\right)}^{3}-1}\right)\;+{\left(\nu -1\right)}^{2}\left(\frac{{\left(\nu -1\right)}^{(d-2)+2}-1}{{\left(\nu -1\right)}^{3}-1}\right)\;=\frac{h({\left(\nu -1\right)}^{d+2}-{\left(\nu -1\right)}^{2})}{{\left(\nu -1\right)}^{3}-1}+\left\lceil \frac{h-4}{3}\right\rceil ,$$

and

$$\mathrm{reg}\;\left(Z/(I(T_{\nu ,d,h}),z_{h-1}^{\left(0\right)})\right)=\mathrm{reg}(Q_{\nu ,d,h-2})+\mathrm{reg}(Q_{\nu ,d-1,1}^{\left(\nu -1\right)})+\mathrm{reg}(Q_{\nu ,d,1})\;=\mathrm{reg}(Q_{\nu ,d,h-2})+(\nu -1)\mathrm{reg}(Q_{\nu ,d-1,1})+\mathrm{reg}(Q_{\nu ,d,1})\;=(h-2)\left(\frac{{\left(\nu -1\right)}^{d+2}-{\left(\nu -1\right)}^{2}}{{\left(\nu -1\right)}^{3}-1}\right)+\left\lceil \frac{h-3}{3}\right\rceil \;+(\nu -1)\left(\frac{{\left(\nu -1\right)}^{(d-1)+2}-(\nu -1)}{{\left(\nu -1\right)}^{3}-1}\right)+\frac{{\left(\nu -1\right)}^{d+2}-{\left(\nu -1\right)}^{2}}{{\left(\nu -1\right)}^{3}-1}\;=\frac{h({\left(\nu -1\right)}^{d+2}-{\left(\nu -1\right)}^{2})}{{\left(\nu -1\right)}^{3}-1}+\left\lceil \frac{h-3}{3}\right\rceil .$$

If h≡0(mod3), then $\left\lceil \frac{h-4}{3}\right\rceil =\left\lceil \frac{h-3}{3}\right\rceil$, so we have

$$\mathrm{reg}\;\left(Z/(I(T_{\nu ,d,h}),z_{h-1}^{\left(0\right)})\right)=\mathrm{reg}\;\left(Z/(I(T_{\nu ,d,h}):z_{h-1}^{\left(0\right)})\right).$$

Thus, by Lemma 2.6 (*b*), we have

$$\mathrm{reg}(Q_{\nu ,d,h})\in \left\{\mathrm{reg}\;\left(Z/(I(T_{\nu ,d,h}),z_{h-1}^{\left(0\right)})\right)+1,\mathrm{reg}\;\left(Z/(I(T_{\nu ,d,h}),z_{h-1}^{\left(0\right)})\right)\right\}\;\;\mathrm{reg}(Q_{\nu ,d,h})\in \left\{\frac{h({\left(\nu -1\right)}^{d+2}-{\left(\nu -1\right)}^{2})}{{\left(\nu -1\right)}^{3}-1}+\left\lceil \frac{h-4}{3}\right\rceil +1,\frac{h({\left(\nu -1\right)}^{d+2}-{\left(\nu -1\right)}^{2})}{{\left(\nu -1\right)}^{3}-1}+\left\lceil \frac{h-4}{3}\right\rceil \right\}\;\;\mathrm{reg}(Q_{\nu ,d,h})\leq \frac{h({\left(\nu -1\right)}^{d+2}-{\left(\nu -1\right)}^{2})}{{\left(\nu -1\right)}^{3}-1}+\left\lceil \frac{h-1}{3}\right\rceil .$$

If h≡1(mod3), then $\left\lceil \frac{h-3}{3}\right\rceil =\left\lceil \frac{h-1}{3}\right\rceil$, so we have

$$\mathrm{reg}\;\left(Z/(I(T_{\nu ,d,h}),z_{h-1}^{\left(0\right)})\right)>\mathrm{reg}\;\left(Z/(I(T_{\nu ,d,h}):z_{h-1}^{\left(0\right)})\right).$$

Thus, by using Lemma 2.2 (*c*), $\mathrm{reg}(Q_{\nu ,d,h})=\frac{h({\left(\nu -1\right)}^{d+2}-{\left(\nu -1\right)}^{2})}{{\left(\nu -1\right)}^{3}-1}+\left\lceil \frac{h-1}{3}\right\rceil$. If h≡2(mod3), then $\left\lceil \frac{h-4}{3}\right\rceil =\left\lceil \frac{h-3}{3}\right\rceil$, so we have

$$\mathrm{reg}\;\left(Z/(I(T_{\nu ,d,h}),z_{h-1}^{\left(0\right)})\right)=\mathrm{reg}\;\left(Z/(I(T_{\nu ,d,h}):z_{h-1}^{\left(0\right)})\right).$$

Thus, by using Lemma 2.2 (*b*), we have

$$\mathrm{reg}(Q_{\nu ,d,h})\in \left\{\mathrm{reg}\;\left(Z/(I(T_{\nu ,d,h}),z_{h-1}^{\left(0\right)})\right)+1,\mathrm{reg}\;\left(Z/(I(T_{\nu ,d,h}),z_{h-1}^{\left(0\right)})\right)\right\}\;\;\mathrm{reg}(Q_{\nu ,d,h})\in \left\{\frac{h({\left(\nu -1\right)}^{d+2}-{\left(\nu -1\right)}^{2})}{{\left(\nu -1\right)}^{3}-1}+\left\lceil \frac{h-4}{3}\right\rceil +1,\frac{h({\left(\nu -1\right)}^{d+2}-{\left(\nu -1\right)}^{2})}{{\left(\nu -1\right)}^{3}-1}+\left\lceil \frac{h-4}{3}\right\rceil \right\}\;\;\mathrm{reg}(Q_{\nu ,d,h})\leq \frac{h({\left(\nu -1\right)}^{d+2}-{\left(\nu -1\right)}^{2})}{{\left(\nu -1\right)}^{3}-1}+\left\lceil \frac{h-1}{3}\right\rceil .$$

Now for finding lower bound when h≡0,2(mod3) we find an induced matching of $T_{\nu ,d,h}$ as follows:

(3.7) $$E(d-1,d)=\overset{h{\left(\nu -1\right)}^{d-1}}{\underset{i=1}{⋃}}\left\{\{z_{i}^{\left(d-1\right)},z_{(\nu -1)i}^{\left(d\right)}\}\right\},$$

where $|E(d-1,d)|=h{\left(\nu -1\right)}^{d-1}$ and let E=E(d−1,d)∪E(d−4,d−3)∪…∪E(2,3). Let 1≤j≤h−1, $I_{j}=\left\{\{z_{j}^{\left(0\right)},z_{j+1}^{\left(0\right)}\}\right\}$ be the edges of graph $T_{2,d,h}$. Let $I=\left\{I_{1},I_{4},\ldots ,I_{1+3\left\lceil \frac{h-4}{3}\right\rceil }\right\}$ and M=E∪I. It can easily be verified that *M* is an induced matching. Therefore, $\mathrm{indmat}(T_{\nu ,d,h})\geq |M|$, where $|M|=h{\left(\nu -1\right)}^{d-1}+h{\left(\nu -1\right)}^{d-4}+\cdots +h{\left(\nu -1\right)}^{2}+\left\lceil \frac{h-1}{3}\right\rceil =\frac{h({\left(\nu -1\right)}^{d+2}-{\left(\nu -1\right)}^{2})}{{\left(\nu -1\right)}^{3}-1}+\left\lceil \frac{h-1}{3}\right\rceil$. According to Lemma 2.5, we have $\mathrm{reg}(Q_{\nu ,d,h})\geq \frac{h({\left(\nu -1\right)}^{d+2}-{\left(\nu -1\right)}^{2})}{{\left(\nu -1\right)}^{3}-1}+\left\lceil \frac{h-1}{3}\right\rceil$. Hence, $\mathrm{reg}(Q_{\nu ,d,h})=\frac{h({\left(\nu -1\right)}^{d+2}-{\left(\nu -1\right)}^{2})}{{\left(\nu -1\right)}^{3}-1}+\left\lceil \frac{h-1}{3}\right\rceil$.

**Case 3 :** If d≡1(mod3). As d−2≡2(mod3) and d−1≡0(mod3), for h=2, using Remark 3.2, Lemma 3.4, Lemma 2.8 and Equations (3.3), (3.4) we have

$$\mathrm{reg}\;\left(Z/(I(T_{\nu ,d,2}):z_{1}^{\left(0\right)})\right)=\mathrm{reg}(Q_{\nu ,d-2,1}^{{\left(\nu -1\right)}^{2}})+\mathrm{reg}(Q_{\nu ,d-1,1}^{\left(\nu -1\right)})+\mathrm{reg}(K[z_{1}^{\left(0\right)}])\;={\left(\nu -1\right)}^{2}\left(\frac{{\left(\nu -1\right)}^{(d-2)+2}-(\nu -1)}{{\left(\nu -1\right)}^{3}-1}\right)\;+(\nu -1)\left(\frac{{\left(\nu -1\right)}^{(d-1)+2}-{\left(\nu -1\right)}^{2}}{{\left(\nu -1\right)}^{3}-1}\right)\;=\frac{{\left(\nu -1\right)}^{d+2}-{\left(\nu -1\right)}^{3}}{{\left(\nu -1\right)}^{3}-1}+\frac{{\left(\nu -1\right)}^{d+2}-{\left(\nu -1\right)}^{3}}{{\left(\nu -1\right)}^{3}-1}+2-2\;=\frac{2({\left(\nu -1\right)}^{d+2}-1)}{{\left(\nu -1\right)}^{3}-1}-2,$$

and

$$\mathrm{reg}\;\left(Z/(I(T_{\nu ,d,2}),z_{1}^{\left(0\right)})\right)=\mathrm{reg}(Q_{\nu ,d-1,1}^{\left(\nu -1\right)})+\mathrm{reg}(Q_{\nu ,d,1})\;=(\nu -1)\left(\frac{{\left(\nu -1\right)}^{(d-1)+2}-{\left(\nu -1\right)}^{2}}{{\left(\nu -1\right)}^{3}-1}\right)+\frac{{\left(\nu -1\right)}^{d+2}-1}{{\left(\nu -1\right)}^{3}-1}\;=\frac{{\left(\nu -1\right)}^{d+2}-{\left(\nu -1\right)}^{3}}{{\left(\nu -1\right)}^{3}-1}+1+\frac{{\left(\nu -1\right)}^{d+2}-1}{{\left(\nu -1\right)}^{3}-1}-1\;=\frac{2({\left(\nu -1\right)}^{d+2}-1)}{{\left(\nu -1\right)}^{3}-1}-1.$$

Now for h≥3, using Remark 3.2, Lemma 3.4, Lemma 2.8 and Equations (3.5), (3.6), and applying induction on *h* we have

$$\mathrm{reg}(Z/(I(T_{\nu ,d,h}):z_{h-1}^{\left(0\right)}))\;=\mathrm{reg}(Q_{\nu ,d,h-3})+\sum_{i=1}^{2}\mathrm{reg}(Q_{\nu ,d-1,1}^{\left(\nu -1\right)})+\mathrm{reg}(Q_{\nu ,d-2,1}^{{\left(\nu -1\right)}^{2}})+\mathrm{reg}(K[z_{h-1}^{\left(0\right)}])\;=\mathrm{reg}(Q_{\nu ,d,h-3})+\sum_{i=1}^{2}(\nu -1)\mathrm{reg}(Q_{\nu ,d-1,1})+{\left(\nu -1\right)}^{2}\mathrm{reg}(Q_{\nu ,d-2,1})+0\;=(h-3)\left(\frac{{\left(\nu -1\right)}^{d+2}-1}{{\left(\nu -1\right)}^{3}-1}\right)-\left\lfloor \frac{h-3}{2}\right\rfloor +2(\nu -1)\left(\frac{{\left(\nu -1\right)}^{(d-1)+2}-{\left(\nu -1\right)}^{2}}{{\left(\nu -1\right)}^{3}-1}\right)\;+{\left(\nu -1\right)}^{2}\left(\frac{{\left(\nu -1\right)}^{(d-2)+2}-(\nu -1)}{{\left(\nu -1\right)}^{3}-1}\right)\;=(h-3)\left(\frac{{\left(\nu -1\right)}^{d+2}-1}{{\left(\nu -1\right)}^{3}-1}\right)-\left\lfloor \frac{h-3}{2}\right\rfloor +\frac{3({\left(\nu -1\right)}^{d+2}-{\left(\nu -1\right)}^{3})}{{\left(\nu -1\right)}^{3}-1}+3-3\;=\frac{h({\left(\nu -1\right)}^{d+2}-1)}{{\left(\nu -1\right)}^{3}-1}-\left\lfloor \frac{h-3}{2}\right\rfloor -3\;=\frac{h({\left(\nu -1\right)}^{d+2}-1)}{{\left(\nu -1\right)}^{3}-1}-\left\lfloor \frac{h+3}{2}\right\rfloor ,$$

and

$$\mathrm{reg}(Z/(I(T_{\nu ,d,h}),z_{h-1}^{\left(0\right)}))\;=\mathrm{reg}(Q_{\nu ,d,h-2})+\mathrm{reg}(Q_{\nu ,d-1,1}^{\left(\nu -1\right)})+\mathrm{reg}(Q_{\nu ,d,1})\;=\mathrm{reg}(Q_{\nu ,d,h-2})+(\nu -1)\mathrm{reg}(Q_{\nu ,d-1,1})+\mathrm{reg}(Q_{\nu ,d,1})\;=(h-2)\left(\frac{{\left(\nu -1\right)}^{d+2}-1}{{\left(\nu -1\right)}^{3}-1}\right)-\left\lfloor \frac{h-2}{2}\right\rfloor +(\nu -1)\left(\frac{{\left(\nu -1\right)}^{(d-1)+2}-{\left(\nu -1\right)}^{2}}{{\left(\nu -1\right)}^{3}-1}\right)\;+\frac{{\left(\nu -1\right)}^{d+2}-1}{{\left(\nu -1\right)}^{3}-1}\;=(h-1)\left(\frac{{\left(\nu -1\right)}^{d+2}-1}{{\left(\nu -1\right)}^{3}-1}\right)-\left\lfloor \frac{h-2}{2}\right\rfloor +\frac{{\left(\nu -1\right)}^{d+2}-{\left(\nu -1\right)}^{3}}{{\left(\nu -1\right)}^{3}-1}+1-1\;=\frac{h({\left(\nu -1\right)}^{d+2}-1)}{{\left(\nu -1\right)}^{3}-1}-\left\lfloor \frac{h-2}{2}\right\rfloor -1\;=\frac{h({\left(\nu -1\right)}^{d+2}-1)}{{\left(\nu -1\right)}^{3}-1}-\left\lfloor \frac{h}{2}\right\rfloor .$$

Clearly for all h≥2, $\mathrm{reg}\;\left(Z/(I(T_{\nu ,d,h}),z_{h-1}^{\left(0\right)})\right)>\mathrm{reg}\;\left(Z/(I(T_{\nu ,d,h}):z_{h-1}^{\left(0\right)})\right)$. Hence, by Lemma 2.6(*c*), we have,

$$\mathrm{reg}(Q_{\nu ,d,h})=\frac{h({\left(\nu -1\right)}^{d+2}-1)}{{\left(\nu -1\right)}^{3}-1}-\left\lfloor \frac{h}{2}\right\rfloor .$$

 □

Theorem 3.6 *Let* d≥1 *,* h≥3 *and* $Z=K[V(C_{2,d,h})]$ *. Then*

$$\mathrm{reg}(Z/I(C_{2,d,h}))=\left\{ \begin{array}{cc} \frac{hd}{3}+\left\lceil \frac{h-1}{3}\right\rceil , & d\equiv 0(\mathrm{mod}\;3);\\ h\left\lceil \frac{d}{3}\right\rceil -\left\lfloor \frac{h+1}{2}\right\rfloor , & d\equiv 1(\mathrm{mod}\;3);\\ h\left\lceil \frac{d}{3}\right\rceil , & d\equiv 2(\mathrm{mod}\;3). \end{array}\right.$$

Proof If d=1, the result follows from Lemma 2.3, $\mathrm{reg}(Z/I(C_{2,1,h}))=\left\lceil \frac{h-1}{2}\right\rceil =h-\left\lfloor \frac{h+1}{2}\right\rfloor$. Now let d≥2, we have the following isomorphisms:

(3.8) $$Z/(I(C_{2,d,h}):z_{h}^{\left(0\right)})≅Q_{2,d,h-3}{⊗}_{K}\underset{i=1}{\overset{2}{{⊗}_{K}}}K[V(P_{d})]/I(P_{d}){⊗}_{K}K[V(P_{d-1})]/I(P_{d-1}){⊗}_{K}K[z_{h}^{\left(0\right)}],\;$$

and

(3.9) $$Z/(I(C_{2,d,h}),z_{h}^{\left(0\right)})≅Q_{2,d,h-1}{⊗}_{K}K[V(P_{d})]/I(P_{d}).\;$$

**Case 1 :** Let d≡2(mod3). Using Lemma 2.8, Lemma 2.4, Theorem 3.3 and Equations (3.8), (3.9) we have

$$\mathrm{reg}\;\left(Z/(I(C_{2,d,h}):z_{h}^{\left(0\right)})\right)=\mathrm{reg}(Q_{2,d,h-3})+\sum_{i=1}^{2}\mathrm{reg}(K[V(P_{d})]/I(P_{d}))+\mathrm{reg}(K[V(P_{d-1})]/I(P_{d-1}))\;+\mathrm{reg}(K[z_{h}^{\left(0\right)}])\;=(h-3)\left\lceil \frac{d}{3}\right\rceil +2\left\lceil \frac{d-1}{3}\right\rceil +\left\lceil \frac{d-2}{3}\right\rceil \;=(h-3)\left\lceil \frac{d}{3}\right\rceil +2\left\lceil \frac{d}{3}\right\rceil +\left(\left\lceil \frac{d}{3}\right\rceil -1\right)\;=h\left\lceil \frac{d}{3}\right\rceil -1,$$

and

$$\mathrm{reg}\;\left(Z/(I(C_{2,d,h}),z_{h}^{\left(0\right)})\right)=\mathrm{reg}(Q_{2,d,h-1})+\mathrm{reg}(K[V(P_{d})]/I(P_{d}))\;=(h-1)\left\lceil \frac{d}{3}\right\rceil +\left\lceil \frac{d-1}{3}\right\rceil \;=(h-1)\left\lceil \frac{d}{3}\right\rceil +\left\lceil \frac{d}{3}\right\rceil \;=h\left\lceil \frac{d}{3}\right\rceil .$$

Clearly, $\mathrm{reg}\;\left(Z/(I(C_{2,d,h}),z_{h}^{\left(0\right)})\right)>\mathrm{reg}\;\left(Z/(I(C_{2,d,h}):z_{h}^{\left(0\right)})\right)$. Hence, by Lemma 2.6 (*c*),

$$\mathrm{reg}(Z/I(C_{2,d,h}))=h\left\lceil \frac{d}{3}\right\rceil .$$

**Case 2 :** Let d≡0(mod3). Using Lemma 2.8, Lemma 2.4, Theorem 3.3 and Equations (3.8), (3.9) we have

$$\mathrm{reg}\;\left(Z/(I(C_{2,d,h}):z_{h}^{\left(0\right)})\right)=\mathrm{reg}(Q_{2,d,h-3})+\sum_{i=1}^{2}\mathrm{reg}(K[V(P_{d})]/I(P_{d}))+\mathrm{reg}(K[V(P_{d-1})]/I(P_{d-1}))\;+\mathrm{reg}(K[z_{h}^{\left(0\right)}])\;=(h-3)\frac{d}{3}+\left\lceil \frac{h-4}{3}\right\rceil +2\left\lceil \frac{d-1}{3}\right\rceil +\left\lceil \frac{d-2}{3}\right\rceil ,$$

and

$$\mathrm{reg}\;\left(Z/(I(C_{2,d,h}),z_{h}^{\left(0\right)})\right)=\mathrm{reg}(Q_{2,d,h-1})+\mathrm{reg}(K[V(P_{d})]/I(P_{d}))\;=(h-1)\frac{d}{3}+\left\lceil \frac{h-2}{3}\right\rceil +\left\lceil \frac{d-1}{3}\right\rceil .$$

Since d≡0(mod3), $\left\lceil \frac{d-2}{3}\right\rceil =\left\lceil \frac{d-1}{3}\right\rceil =\frac{d}{3}$. Thus, we have

$$\mathrm{reg}\;\left(Z/(I(C_{2,d,h}),z_{h}^{\left(0\right)})\right)=\frac{hd}{3}+\left\lceil \frac{h-2}{3}\right\rceil ,$$

and

$$\mathrm{reg}\;\left(Z/(I(C_{2,d,h}):z_{h}^{\left(0\right)})\right)=\frac{hd}{3}+\left\lceil \frac{h-4}{3}\right\rceil .$$

If h≡0,1(mod3), then $\left\lceil \frac{h-2}{3}\right\rceil =\left\lceil \frac{h-1}{3}\right\rceil$ and $\left\lceil \frac{h-1}{3}\right\rceil >\left\lceil \frac{h-4}{3}\right\rceil$. So we have

$$\mathrm{reg}\;\left(Z/(I(C_{2,d,h}),z_{h}^{\left(0\right)})\right)>\mathrm{reg}\;\left(Z/(I(C_{2,d,h}):z_{h}^{\left(0\right)})\right).$$

Hence, by Lemma 2.6, $\mathrm{reg}(Z/I(C_{2,d,h}))=\frac{hd}{3}+\left\lceil \frac{h-1}{3}\right\rceil$.

If h≡2(mod3), then $\left\lceil \frac{h-4}{3}\right\rceil =\left\lceil \frac{h-2}{3}\right\rceil$, so we have $\mathrm{reg}\;\left(Z/(I(C_{2,d,h}),z_{h}^{\left(0\right)})\right)=\mathrm{reg}\;\left(Z/(I(C_{2,d,h}):z_{h}^{\left(0\right)})\right)$. Thus, by using Lemma 2.6 (*b*), we have

$$\mathrm{reg}(Z/I(C_{2,d,h}))\in \left\{\mathrm{reg}\;\left(Z/(I(C_{2,d,h}),z_{h}^{\left(0\right)})\right)+1,\mathrm{reg}\;\left(Z/(I(C_{2,d,h}),z_{h}^{\left(0\right)})\right)\right\}\;\;\mathrm{reg}(Z/I(C_{2,d,h}))\in \left\{\frac{hd}{3}+\left\lceil \frac{h-2}{3}\right\rceil +1,\frac{hd}{3}+\left\lceil \frac{h-2}{3}\right\rceil \right\}\;\;\mathrm{reg}(Z/I(C_{2,d,h}))\leq \frac{hd}{3}+\left\lceil \frac{h-2}{3}\right\rceil +1.$$

Now for finding lower bound when h≡2(mod3) we find an induced matching of $C_{2,d,h}$ as follows:

$$E_{1}=\overset{h}{\underset{i=1}{⋃}}\left\{\{z_{i}^{\left(d-1\right)},z_{i}^{\left(d\right)}\}\right\},E_{2}=\overset{h}{\underset{i=1}{⋃}}\left\{\{z_{i}^{\left(d-4\right)},z_{i}^{\left(d-3\right)}\}\right\},\cdots ,E_{\frac{d}{3}}=\overset{h}{\underset{i=1}{⋃}}\left\{\{z_{i}^{\left(2\right)},z_{i}^{\left(3\right)}\}\right\}.$$

Clearly $|E_{i}|=h$, for all *i*. Let 1≤j≤h−1, $I_{j}=\left\{\{z_{j}^{\left(0\right)},z_{j+1}^{\left(0\right)}\}\right\}$ be the edges of graph $C_{2,d,h}$. Let $I=\left\{I_{1},I_{4},\ldots ,I_{1+3\left\lceil \frac{h-5}{3}\right\rceil }\right\}$ and $E=E_{1}\cup E_{2}\cup \ldots \cup E_{\frac{d}{3}}\cup I$. It can easily be verified that *E* is an induced matching, where $|E|=\frac{hd}{3}+\left\lceil \frac{h-2}{3}\right\rceil$. Thus, by using Theorem 2.7$,\mathrm{reg}(Z/I(C_{2,d,h}))=\mathrm{indmat}(C_{2,d,h})+1\geq |E|+1=\frac{hd}{3}+\left\lceil \frac{h-2}{3}\right\rceil +1=\frac{hd}{3}+\left\lceil \frac{h+1}{3}\right\rceil =\frac{hd}{3}+\left\lceil \frac{h-1}{3}\right\rceil$. Hence, $\mathrm{reg}(Z/I(C_{2,d,h}))=\frac{hd}{3}+\left\lceil \frac{h-1}{3}\right\rceil$.

**Case 3 :** Let d≡1(mod3). Using Lemma 2.8, Lemma 2.4, Theorem 3.3 and Equations (3.8), (3.9) we have

$$\mathrm{reg}\;\left(Z/(I(C_{2,d,h}):z_{h}^{\left(0\right)})\right)=\mathrm{reg}(Q_{2,d,h-3})+\sum_{i=1}^{2}\mathrm{reg}(K[V(P_{d})]/I(P_{d}))+\mathrm{reg}(K[V(P_{d-1})]/I(P_{d-1}))\;+\mathrm{reg}(K[z_{h}^{\left(0\right)}])\;=(h-3)\left\lceil \frac{d}{3}\right\rceil -\left\lfloor \frac{h-3}{2}\right\rfloor +2\left\lceil \frac{d-1}{3}\right\rceil +\left\lceil \frac{d-2}{3}\right\rceil \;=(h-3)\left\lceil \frac{d}{3}\right\rceil -\left\lfloor \frac{h-3}{2}\right\rfloor +2\left(\left\lceil \frac{d}{3}\right\rceil -1\right)+\left(\left\lceil \frac{d}{3}\right\rceil -1\right)\;=h\left\lceil \frac{d}{3}\right\rceil -\left(\left\lfloor \frac{h-3}{2}\right\rfloor +3\right)\;=h\left\lceil \frac{d}{3}\right\rceil -\left\lfloor \frac{h+3}{2}\right\rfloor ,$$

and

$$\mathrm{reg}\;\left(Z/(I(C_{2,d,h}),z_{h}^{\left(0\right)})\right)=\mathrm{reg}(Q_{2,d,h-1})+\mathrm{reg}(K[V(P_{d})]/I(P_{d}))\;=(h-1)\left\lceil \frac{d}{3}\right\rceil -\left\lfloor \frac{h-1}{2}\right\rfloor +\left\lceil \frac{d-1}{3}\right\rceil \;=(h-1)\left\lceil \frac{d}{3}\right\rceil -\left\lfloor \frac{h-1}{2}\right\rfloor +\left(\left\lceil \frac{d}{3}\right\rceil -1\right)\;=h\left\lceil \frac{d}{3}\right\rceil -\left\lfloor \frac{h+1}{2}\right\rfloor .$$

Clearly, $\mathrm{reg}\;\left(Z/(I(C_{2,d,h}),z_{h}^{\left(0\right)})\right)>\mathrm{reg}\;\left(Z/(I(C_{2,d,h}):z_{h}^{\left(0\right)})\right)$. Hence, by Lemma 2.6 (*c*),

$$\mathrm{reg}(Z/I(C_{2,d,h}))=h\left\lceil \frac{d}{3}\right\rceil -\left\lfloor \frac{h+1}{2}\right\rfloor .$$

 □

Theorem 3.7 *Let* ν,h≥3 *and* d≥1 *. If* $Z=K[V(C_{\nu ,d,h})]$ *, then*

$$\mathrm{reg}(Z/I(C_{\nu ,d,h}))=\left\{ \begin{array}{cc} \frac{h({\left(\nu -1\right)}^{d+2}-{\left(\nu -1\right)}^{2})}{{\left(\nu -1\right)}^{3}-1}+\left\lceil \frac{h-1}{3}\right\rceil , & d\equiv 0(\mathrm{mod}\;3);\\ \frac{h({\left(\nu -1\right)}^{d+2}-1)}{{\left(\nu -1\right)}^{3}-1}-\left\lfloor \frac{h+1}{2}\right\rfloor , & d\equiv 1(\mathrm{mod}\;3);\\ \frac{h({\left(\nu -1\right)}^{d+2}-(\nu -1))}{{\left(\nu -1\right)}^{3}-1}, & d\equiv 2(\mathrm{mod}\;3). \end{array}\right.$$

Proof If d=1, the result follows from Lemma 2.3, $\mathrm{reg}(Z/I(C_{\nu ,d,h}))=\left\lceil \frac{h-1}{2}\right\rceil =h-\left\lfloor \frac{h+1}{2}\right\rfloor$. Let d≥2, we have the following isomorphisms:

(3.10) $$Z/(I(C_{\nu ,d,h}):z_{h}^{\left(0\right)})≅Q_{\nu ,d,h-3}{⊗}_{K}\underset{i=1}{\overset{2}{{⊗}_{K}}}Q_{\nu ,d-1,1}^{\left(\nu -1\right)}{⊗}_{K}Q_{\nu ,d-2,1}^{{\left(\nu -1\right)}^{2}},$$

and

(3.11) $$Z/(I(C_{\nu ,d,h}),z_{h}^{\left(0\right)})≅Q_{\nu ,d,h-1}{⊗}_{K}Q_{\nu ,d-1,1}^{\left(\nu -1\right)}.$$

We consider the following three cases:

**Case 1 :** Let d≡2(mod3). As d−1≡1(mod3) and d−2≡0(mod3), using Remark 3.2, Lemma 3.4, Lemma 2.8 and Equations (3.10), (3.11), and applying induction on *h* we have

$$\mathrm{reg}(Z/(I(C_{\nu ,d,h}):z_{h}^{\left(0\right)}))\;=\mathrm{reg}(Q_{\nu ,d,h-3})+\sum_{i=1}^{2}\mathrm{reg}(Q_{\nu ,d-1,1}^{\left(\nu -1\right)})+\mathrm{reg}(Q_{\nu ,d-2,1}^{{\left(\nu -1\right)}^{2}})\;=(h-3)\left(\frac{{\left(\nu -1\right)}^{d+2}-(\nu -1)}{{\left(\nu -1\right)}^{3}-1}\right)+2(\nu -1)\left(\frac{{\left(\nu -1\right)}^{(d-1)+2}-1}{{\left(\nu -1\right)}^{3}-1}\right)\;+{\left(\nu -1\right)}^{2}\left(\frac{{\left(\nu -1\right)}^{(d-2)+2}-{\left(\nu -1\right)}^{2}}{{\left(\nu -1\right)}^{3}-1}\right)\;=(h-1)\left(\frac{{\left(\nu -1\right)}^{d+2}-(\nu -1)}{{\left(\nu -1\right)}^{3}-1}\right)+\frac{{\left(\nu -1\right)}^{d+2}-{\left(\nu -1\right)}^{4}}{{\left(\nu -1\right)}^{3}-1}+(\nu -1)\;-(\nu -1)\;=\frac{h({\left(\nu -1\right)}^{d+2}-(\nu -1))}{{\left(\nu -1\right)}^{3}-1}-(\nu -1),$$

and

$$\mathrm{reg}\;\left(Z/(I(C_{\nu ,d,h}),z_{h}^{\left(0\right)})\right)=\mathrm{reg}(Q_{\nu ,d,h-1})+\mathrm{reg}(Q_{\nu ,d-1,1}^{\left(\nu -1\right)})\;=(h-1)\left(\frac{{\left(\nu -1\right)}^{d+2}-(\nu -1)}{{\left(\nu -1\right)}^{3}-1}\right)+(\nu -1)\left(\frac{{\left(\nu -1\right)}^{(d-1)+2}-1}{{\left(\nu -1\right)}^{3}-1}\right)\;=\frac{h({\left(\nu -1\right)}^{d+2}-(\nu -1))}{{\left(\nu -1\right)}^{3}-1}.$$

Hence, by Lemma 2.6 (*c*), $\mathrm{reg}(Z/I(C_{\nu ,d,h}))=\frac{h({\left(\nu -1\right)}^{d+2}-(\nu -1))}{{\left(\nu -1\right)}^{3}-1}$.

**Case 2 :** Let d≡0(mod3). As d−2≡1(mod3) and d−1≡2(mod3), using Remark 3.2, Lemma 3.4, Lemma 2.8 and Equations (3.10), (3.11), and applying induction on *h* we have

$$\mathrm{reg}\;\left(Z/(I(C_{\nu ,d,h}):z_{h}^{\left(0\right)})\right)=\mathrm{reg}(Q_{\nu ,d,h-3})+\sum_{i=1}^{2}\mathrm{reg}(Q_{\nu ,d-1,1}^{\left(\nu -1\right)})+\mathrm{reg}(Q_{\nu ,d-2,1}^{{\left(\nu -1\right)}^{2}})\;=(h-3)\left(\frac{{\left(\nu -1\right)}^{d+2}-{\left(\nu -1\right)}^{2}}{{\left(\nu -1\right)}^{3}-1}\right)+\left\lceil \frac{h-4}{3}\right\rceil +2\left(\frac{{\left(\nu -1\right)}^{d+2}-\nu +1}{{\left(\nu -1\right)}^{3}-1}\right)\;+{\left(\nu -1\right)}^{2}\left(\frac{{\left(\nu -1\right)}^{(d-2)+2}-1}{{\left(\nu -1\right)}^{3}-1}\right)\;=\frac{h({\left(\nu -1\right)}^{d+2}-{\left(\nu -1\right)}^{2})}{{\left(\nu -1\right)}^{3}-1}+\left\lceil \frac{h-4}{3}\right\rceil ,$$

and

$$\mathrm{reg}\;\left(Z/(I(C_{\nu ,d,h}),z_{h}^{\left(0\right)})\right)=\mathrm{reg}(Q_{\nu ,d,h-1})+\mathrm{reg}(Q_{\nu ,d-1,1}^{\left(\nu -1\right)})\;=(h-1)\left(\frac{{\left(\nu -1\right)}^{d+2}-{\left(\nu -1\right)}^{2}}{{\left(\nu -1\right)}^{3}-1}\right)+\left\lceil \frac{h-2}{3}\right\rceil \;+(\nu -1)\left(\frac{{\left(\nu -1\right)}^{(d-1)+2}-\nu +1}{{\left(\nu -1\right)}^{3}-1}\right)\;=\frac{h({\left(\nu -1\right)}^{d+2}-{\left(\nu -1\right)}^{2})}{{\left(\nu -1\right)}^{3}-1}+\left\lceil \frac{h-2}{3}\right\rceil .$$

If h≡0,1(mod3), then $\left\lceil \frac{h-2}{3}\right\rceil =\left\lceil \frac{h-1}{3}\right\rceil$ and $\left\lceil \frac{h-1}{3}\right\rceil >\left\lceil \frac{h-4}{3}\right\rceil$. So we have

$$\mathrm{reg}\;\left(Z/(I(C_{\nu ,d,h}),z_{h}^{\left(0\right)})\right)>\mathrm{reg}\;\left(Z/(I(C_{\nu ,d,h}):z_{h}^{\left(0\right)})\right).$$

Hence, by Lemma 2.6(*c*), $\mathrm{reg}(Z/I(C_{\nu ,d,h}))=\frac{h({\left(\nu -1\right)}^{d+2}-{\left(\nu -1\right)}^{2})}{{\left(\nu -1\right)}^{3}-1}+\left\lceil \frac{h-1}{3}\right\rceil$.

If h≡2(mod3), then $\left\lceil \frac{h-4}{3}\right\rceil =\left\lceil \frac{h-2}{3}\right\rceil$, so we have

$$\mathrm{reg}\;\left(Z/(I(C_{\nu ,d,h}),z_{h}^{\left(0\right)})\right)=\mathrm{reg}\;\left(Z/(I(C_{\nu ,d,h}):z_{h}^{\left(0\right)})\right).$$

Thus, by using Lemma 2.6 (*b*), we have

$$\mathrm{reg}(Z/I(C_{\nu ,d,h}))\in \left\{\mathrm{reg}\;\left(Z/(I(C_{\nu ,d,h}),z_{h}^{\left(0\right)})\right)+1,\mathrm{reg}\;\left(Z/(I(C_{\nu ,d,h}),z_{h}^{\left(0\right)})\right)\right\}\;\;\mathrm{reg}(Z/I(C_{\nu ,d,h}))\in \left\{\frac{h({\left(\nu -1\right)}^{d+2}-{\left(\nu -1\right)}^{2})}{{\left(\nu -1\right)}^{3}-1}+\left\lceil \frac{h-2}{3}\right\rceil +1,\frac{h({\left(\nu -1\right)}^{d+2}-{\left(\nu -1\right)}^{2})}{{\left(\nu -1\right)}^{3}-1}+\left\lceil \frac{h-2}{3}\right\rceil \right\}\;\;\mathrm{reg}(Z/I(C_{\nu ,d,h}))\leq \frac{h({\left(\nu -1\right)}^{d+2}-{\left(\nu -1\right)}^{2})}{{\left(\nu -1\right)}^{3}-1}+\left\lceil \frac{h-2}{3}\right\rceil +1.$$

Now for finding lower bound when h≡2(mod3) we find an induced matching of $C_{\nu ,d,h}$ as follows:

(3.12) $$E(d-1,d)=\overset{h{\left(\nu -1\right)}^{\left(d-1\right)}}{\underset{i=1}{⋃}}\left\{\{z_{i}^{\left(d-1\right)},z_{(\nu -1)i}^{\left(d\right)}\}\right\},$$

where $|E(d-1,d)|=h{\left(\nu -1\right)}^{d-1}$ and let E=E(d−1,d)∪E(d−4,d−3)∪…∪E(2,3). Let 1≤j≤h−1, $I_{j}=\left\{\{z_{j}^{\left(0\right)},z_{j+1}^{\left(0\right)}\}\right\}$ be the edges of graph $C_{\nu ,d,h}$. Let $I=\left\{I_{1},I_{4},\ldots ,I_{1+3\left\lceil \frac{h-5}{3}\right\rceil }\right\}$ and M=E∪I. It can easily be verified that *M* is an induced matching, where $|M|=h{\left(\nu -1\right)}^{d-1}+h{\left(\nu -1\right)}^{d-4}+\cdots +h{\left(\nu -1\right)}^{2}+\left\lceil \frac{h-2}{3}\right\rceil =\frac{h({\left(\nu -1\right)}^{d+2}-{\left(\nu -1\right)}^{2})}{{\left(\nu -1\right)}^{3}-1}+\left\lceil \frac{h-2}{3}\right\rceil$. Thus, by using Lemma 2.7$,\mathrm{reg}\;\left(Z/I(C_{\nu ,d,h})\right)=\mathrm{indmat}(C_{\nu ,d,h})+1\geq |M|+1=\frac{h({\left(\nu -1\right)}^{d+2}-{\left(\nu -1\right)}^{2})}{{\left(\nu -1\right)}^{3}-1}+\left\lceil \frac{h-2}{3}\right\rceil +1=\frac{h({\left(\nu -1\right)}^{d+2}-{\left(\nu -1\right)}^{2})}{{\left(\nu -1\right)}^{3}-1}+\left\lceil \frac{h+1}{3}\right\rceil =\frac{h({\left(\nu -1\right)}^{d+2}-{\left(\nu -1\right)}^{2})}{{\left(\nu -1\right)}^{3}-1}+\left\lceil \frac{h-1}{3}\right\rceil$. Hence, $\mathrm{reg}(Z/I(C_{\nu ,d,h}))=\frac{h({\left(\nu -1\right)}^{d+2}-{\left(\nu -1\right)}^{2})}{{\left(\nu -1\right)}^{3}-1}+\left\lceil \frac{h-1}{3}\right\rceil$.

**Case 3 :** Let d≡1(mod3). As d−1≡0(mod3) and d−2≡2(mod3), using Remark 3.2, Lemma 3.4, Lemma 2.8 and Equations (3.10), (3.11), and applying induction on *h* we have

$$\mathrm{reg}\;\left(Z/(I(C_{\nu ,d,h}):z_{h}^{\left(0\right)})\right)=\mathrm{reg}(Q_{\nu ,d,h-3})+\sum_{i=1}^{2}\mathrm{reg}(Q_{\nu ,d-1,1}^{\left(\nu -1\right)})+\mathrm{reg}(Q_{\nu ,d-2,1}^{{\left(\nu -1\right)}^{2}})\;=(h-3)\left(\frac{{\left(\nu -1\right)}^{d+2}-1}{{\left(\nu -1\right)}^{3}-1}\right)-\left\lfloor \frac{h-3}{2}\right\rfloor \;+2(\nu -1)\left(\frac{{\left(\nu -1\right)}^{(d-1)+2}-{\left(\nu -1\right)}^{2}}{{\left(\nu -1\right)}^{3}-1}\right)\;+{\left(\nu -1\right)}^{2}\left(\frac{{\left(\nu -1\right)}^{(d-2)+2}-\nu +1}{{\left(\nu -1\right)}^{3}-1}\right)\;=(h-3)\left(\frac{{\left(\nu -1\right)}^{d+2}-1}{{\left(\nu -1\right)}^{3}-1}\right)-\left\lfloor \frac{h-3}{2}\right\rfloor +3\left(\frac{{\left(\nu -1\right)}^{d+2}-{\left(\nu -1\right)}^{3}}{{\left(\nu -1\right)}^{3}-1}\right)+3-3\;=\frac{h\left({\left(\nu -1\right)}^{d+2}-1\right)}{{\left(\nu -1\right)}^{3}-1}-\left\lfloor \frac{h+3}{2}\right\rfloor ,$$

and

$$\mathrm{reg}\;\left(Z/(I(C_{\nu ,d,h}),z_{h}^{\left(0\right)})\right)=\mathrm{reg}(Q_{\nu ,d,h-1})+\mathrm{reg}(Q_{\nu ,d-1,1}^{\left(\nu -1\right)})\;=(h-1)\left(\frac{{\left(\nu -1\right)}^{d+2}-1}{{\left(\nu -1\right)}^{3}-1}\right)-\left\lfloor \frac{h-1}{2}\right\rfloor \;+(\nu -1)\left(\frac{{\left(\nu -1\right)}^{(d-1)+2}-{\left(\nu -1\right)}^{2}}{{\left(\nu -1\right)}^{3}-1}\right)\;=(h-1)\left(\frac{{\left(\nu -1\right)}^{d+2}-1}{{\left(\nu -1\right)}^{3}-1}\right)-\left\lfloor \frac{h-1}{2}\right\rfloor +\frac{{\left(\nu -1\right)}^{d+2}-{\left(\nu -1\right)}^{3}}{{\left(\nu -1\right)}^{3}-1}+1-1\;=\frac{h({\left(\nu -1\right)}^{d+2}-1)}{{\left(\nu -1\right)}^{3}-1}-\left\lfloor \frac{h+1}{2}\right\rfloor .$$

Clearly, $\mathrm{reg}\;\left(Z/(I(C_{\nu ,d,h}),z_{h}^{\left(0\right)})\right)>\mathrm{reg}\;\left(Z/(I(C_{\nu ,d,h}):z_{h}^{\left(0\right)})\right)$. Hence, by using Lemma 2.6 (*c*), $\mathrm{reg}\;\left(Z/I(C_{\nu ,d,h})\right)=\frac{h({\left(\nu -1\right)}^{d+2}-1)}{{\left(\nu -1\right)}^{3}-1}-\left\lfloor \frac{h+1}{2}\right\rfloor$. □

## CRediT authorship contribution statement

**Fatima Tul Zahra:** Writing – original draft, Software, Investigation. **Muhammad Ishaq:** Writing – review & editing, Visualization, Validation, Supervision, Methodology, Conceptualization. **Sarah Aljohani:** Writing – review & editing, Validation, Funding acquisition.

## Declaration of Competing Interest

The authors declare that they have no known competing financial interests or personal relationships that could have appeared to influence the work reported in this paper.

## Data Availability

Has data associated with your study been deposited into a publicly available repository? No. Has data associated with your study been deposited into a publicly available repository? No data was used for the research described in the article.
